# Analyzing the effects of memory biases and mood disorders on social performance

**DOI:** 10.1038/s41598-020-77715-6

**Published:** 2020-12-01

**Authors:** Nanda Kishore Sreenivas, Shrisha Rao

**Affiliations:** 1 Oracle, Bangalore, India; 2 International Institute of Information Technology - Bangalore, Bangalore, India

**Keywords:** Computational science, Psychology, Human behaviour

## Abstract

Realistic models of decision-making and social interactions, considering the nature of memory and biases, continue to be an area of immense interest. Emotion and mood are a couple of key factors that play a major role in decisions, nature of social interactions, size of the social network, and the level of engagement. Most of the prior work in this direction focused on a single trait, behavior, or bias. However, this work builds an integrated model that considers multiple traits such as loneliness, the drive to interact, the memory, and mood biases in an agent. The agent system comprises of rational, manic, depressed, and bipolar agents. The system is modeled with an interconnected network, and the size of the personal network of each agent is based on its nature. We consider a game of iterated interactions where an agent cooperates based on its past experiences with the other agent. Through simulation, the effects of various biases and comparative performances of agent types is analyzed. Taking the performance of rational agents as the baseline, manic agents do much better, and depressed agents do much worse. The payoffs also exhibit an almost-linear relationship with the extent of mania. It is also observed that agents with stronger memory perform better than those with weaker memory. For rational agents, there is no significant difference between agents with a positive bias and those with a negative bias. Positive bias is linked with higher payoffs in manic and bipolar agents. In depressed agents, negative bias is linked with higher payoffs. In manic agents, an intermediate value of mood dependence offers the highest payoff. But the opposite is seen in depressed agents. In bipolar agents, agents with weak mood dependence perform the best.

## Introduction

Individual and group decision-making and social interactions have been studied for long and continue to remain of interest. Some early models were fairly simplistic^1,2^, and they assumed perfectly rational behavior which is often not the case in reality. Time and again, research in the domains of psychology and behavioral sciences asserts that humans, as well as animals, are subject to a wide array of cognitive biases^3,4^. Later works moved away from the assumption of perfect rationality and started incorporating various cognitive biases into decision models^5–8^. However, the effects of emotion and mood disorders on decisions and interactions still need to be explored.

Although we humans aspire to be rational decision-makers and never let emotion cloud our judgment, such is never really the case^9^. This has been understood for long—the importance of emotion was captured succinctly by Simon^10^:

“Hence, in order to have anything like a complete theory of human rationality, we have to understand what role emotion plays in it.”

A great deal of research has focused on the impact of emotion on memory but the findings have been extremely diverse, with some claiming emotional memories are indelible^11^, while others claim that emotion has no effect whatsoever on memory^12^. Even among those that agree that emotion has some impact on memory, some claim memories associated with positive emotions are stronger^13,14^ and last longer, while others claim the opposite^15^.

Appraisal theory has been used in prior works to quantitatively define emotion, where an agent evaluates the reward with respect to its goal or expectation^16,17^. If the reward obtained is better than the agent’s expectation, positive emotion is induced; and the reverse also holds. The process of storing information for the first time in memory is called *encoding* and this process leaves a *trace* in the memory. The strength and longevity of the trace depend on how well it has been encoded.

Mood has been seen as the summary of recent emotions^18^. While emotions change after each episode, mood maintains a historical context, yet staying temporally relevant. Mood affects the behavior of an individual in terms of reward perception^19^, memory retrieval^20^, and levels of social engagement^21,22^. There is much to be learned about the effects of emotions and mood in decisions and social interactions. Shevchenko^23^ recently wrote:

“Moods in decision research have been ignored for many years, although decisions made in an emotional state are ubiquitous in daily life. The areas of emotion and decision-making research have remained mostly unfamiliar.”

Mood disorders are prevalent in humans, and studies indicate that about 20% of U.S adults experience some class of mood disorder in their lifetimes^24^. There have been many computational models of various mood disorders^19,25–28^, but most of these assume independent agents without any social interactions or social networks. The extent and frequency of social interactions are intricately linked to mood disorders, with studies indicating that chronically depressed people have smaller social networks than healthy controls^29,30^. Empirical studies also conclude that depressed phases are connected with lower energy, interest, and drive^31,32^. Prior models do not take this into account.

There are several models of mood fluctuation in bipolar disorder such as rhythmic models, discrete models, and models based on the Behavioral Approach System (BAS)^33–37^.

Symptoms of depression include persistent low mood or sadness, and an inability or diminished ability to experience pleasurable stimuli. Depression and mania have been modeled using the concept of *reward sensitivity* in some prior works^19,26,27^. In these models, the reward as perceived by an agent is the product of its reward sensitivity and the actual reward. Depressed agents are assigned values of reward sensitivity between 0 and 1, while manic agents have values greater than 1.

Mood also affects memory retrieval; *mood dependence* is the phenomenon where past events whose emotional state match the current mood are more likely to be retrieved. There seems to be no prior model capturing this effect of mood on memory.

In some works on decision-making, memory is assumed to be infinitely long and perfect^5,38^. However, this is not justified^39,40^, and memories are susceptible to forgetting at various rates. The likelihood of remembering recent events is higher when compared with older events, which is termed the “recency effect”^41,42^. While it seems intuitive that stronger memory enhances performance, some have also argued that forgetting is a useful feature because it helps optimize decision-making^43^. The most common approaches to model forgetting involve the use of exponential decay or power law functions^44–46^.

This work is broadly in the field of computational social science using agent-based modeling^47^, and specifically uses an agent-based model carefully constructed in line with studies in psychology. It is well known that agent-based modeling is a powerful, relatively new tool in the study of social psychology^48^, and that while we cannot yet directly derive outcomes that can guide treatments, it does permit new insights that are not possible using mathematical models and clinical experiments^49^.

We give an integrated model of decisions and social interactions that takes into account the roles of emotion, mood, and memory biases. A game of iterated interactions based on the Continuous Prisoners’ Dilemma (CPD) is considered. As opposed to a standard Iterated Prisoners’ Dilemma which offers only two choices, CPD allows agents to cooperate at various levels between 0 and 1. To explain one such interaction, assume an agent *A* is paired with one of its neighbors *B*. *A* appraises the reward, i.e., the cooperation extended by *B*, with respect to its expectation, and this determines the emotion induced by this interaction. Both agents update their memory and mood, and this affects their subsequent interactions. Agents also broadcast the gist of their interaction to their neighbors.

In our work, emotion is modeled in line with the appraisal theory. To model the effect of emotion on memory, we define the initial strength of a trace as a linear function of emotion. We use two different gradients for positive and negative emotions, allowing us to model both positively and negatively biased agents.

The mood dependence effect is modeled as a probabilistic retrieval of traces using a triangular distribution centered around the current mood of the agent. This ensures traces with similar emotional states have higher chances of being retrieved than others, which is the condition of mood dependence. Forgetting is modeled using exponential decay, in line with prior work^45,46^.

Agents with mood disorders such as mania, depression, and bipolarity are considered in addition to perfectly rational agents. Such an agent society is modeled with an interconnected social network, where agents interact only with their neighbors, and agents broadcast their experiences to their neighbors. Also, the size of the personal network of each agent is determined by its type, which helps fill one significant gap in many prior models that consider agents in isolation.

Agents are also modeled with different levels of engagement or drive. In each round of interaction, an agent has the choice to decide if it wants to participate. Manic agents are modeled with a higher drive and depressed agents with a lower drive, as is consistent with clinical observations^50,51^.

Through simulation, we obtain results about the comparative performances of different types. An agent society with agents of all four types—rational, manic, depressed, and bipolar—is considered. Considering the average payoff of rational agents as the baseline, bipolar agents perform better by roughly 10%. The average payoff of manic agents is approximately 70% higher, and it is about 60% lower for depressed agents. Average payoff is plotted against the extent of mania and depression, and an almost-linear relationship is observed in both cases.

With regard to memory, agents with stronger memory perform better than those with weaker memory. The results differ across types with regard to emotional bias. For rational agents, there is no significant difference between agents with a positive bias and those with a negative bias. In manic agents as well as bipolar agents, positive bias is linked with higher payoff, but the effect is larger for bipolar agents when compared to manic agents. However, among depressed agents, those with a negative bias have higher payoffs.

In the context of mood-dependent retrieval also, the effects vary across different types. For rational agents, there is no significant change in payoff observed by varying the level of mood dependence. In manic agents, an intermediate value of mood dependence offers the highest payoff. But in depressed agents, a similar medium value offers the least payoff. In bipolar agents, agents with weak mood dependence perform the best.

## Background

In this section, we summarize related work in the fields of psychology, decision and behavioral sciences. Each subsection deals with one key aspect of the context of our model.

### Forgetting

Forgetting is the time decay of strength of memory traces in the absence of refreshing, until it is ultimately deleted. This is a fairly common phenomenon in humans, and has a considerable impact in decision-making. The earliest work on memory and forgetting can be traced back to 1885, when Ebbinghaus examined the decline of memory strength with time. Broadly speaking, there have been two theories to model forgetting, on the basis of decay and interference^52–54^.

While both theories agree that old memories are lost over time, decay theory posits time as the only driving force behind forgetting^55,56^. Recent models based on time decay include the primacy model^57^ and the positional decay model^58^. Interference-based models argue that other activities interfere with retrieval of memory items, and thus contribute to forgetting. Typically, these models use an associative network that binds items to contexts, and when new information is encoded it typically overwrites the old, which is thus forgotten^59^.

Rubin and Wenzel considered two hundred datasets and about a hundred different functions, and observed that logarithmic, exponential, hyperbolic and power functions offered the best fit to model forgetting^60^. Since then, exponential decay^44–46^ and power functions^61–63^ seem to be the most favored functions to model forgetting.

### Emotions and memory

Levine and Pizarro write^64^: “Converging evidence from autobiographical memory studies, animal and human laboratory studies, and brain imaging studies shows that emotional events are remembered better than non-emotional events and that mechanisms specific to emotion underlie these effects.” Several other studies also concur with the idea that emotions improve memory for details central to the event and diminish memory for any peripheral details^65,66^.

In the domain of modeling emotions, one of the most influential ideas is the appraisal theory in psychology, where emotions are based on an individual’s evaluation of an interaction with respect to a goal^16,17^. There have been attempts to relate the emotional valence and strength of autobiographical memories, but the findings are varied, with some claiming that positive events are remembered better^13,14^, while others support better memory for negative events^15^.

Negativity bias has been described as the phenomenon by which a negative event has a greater psychological effect even when it is of equal intensity as a positive event^67^. This bias has been found to be fairly ubiquitous in humans^68,69^. Neurological studies also point to greater processing of negative information, which causes asymmetry in attention and memory^70^. In studies concerning positive and negative behaviors, participants tend to recall negative behaviors more than positive behaviors^71,72^.

### Mood and memory

It has been known for long that mood and memory are related, and that the current mood affects which memories are retrieved^73,74^. As Lewis et al.^20^ write, “Remembering all of the negative events of our past lives when depressed is an example of this.” Mood dependence is the phenomenon where retrieval is facilitated for memories that match the present mood. Neuroimaging studies examining the influence of emotion in encoding and retrieval also provide evidence in support of this^75,76^. Further, recent empirical studies based on human participants also offer support to this theory^77^. However, there seem to be no computational models of this phenomenon.

### Types of mood disorders

#### Bipolar disorder

Bipolar disorder^78^ (BP) is a mental disorder characterized by cycling between manic and depressed episodes. Its prevalence is estimated to be 3.3% in the US^79^, and it is thought to affect about 2.4% of the global adult population^80^.

There are several prior computational models of BP. Rhythmic models are based on the idea that there is an intrinsic oscillation of mood, independent of any external perturbation^33,34,81^. These models assume mood swings periodically between mania and depression^82,83^. Another class of models assume multistability, claiming mood tends to distinct discrete states^35,84^. The other popular theory is that the hypersensitivity of the Behavioral Approach System (BAS), and the resulting interactions between mood, expectation, and behavior can explain BP^36,37,85^.

However, among these classes of models, rhythmic models are thought to have close links to actual biological rhythms, such as the circadian rhythm^86^. Indeed, the standard clinical treatment for BP, using Lithium, works through influencing the circadian pathways^87^.

#### Depression and mania

Anhedonia is one of the major symptoms of depression, and it refers to an inability or a diminished ability to experience pleasurable stimuli^51,88^. This model is supported by an empirical study as well, in which self-reported anhedonia was positively correlated with participants’ estimated reward sensitivity^89^. Mania is characterised by elevated mood, energy, and reward-seeking behavior or drive^90^.

A popular approach to model mania and depression is to use reward sensitivity. The perceived reward is determined by the product of actual reward and reward sensitivity^19^; to model mania, reward sensitivity is chosen as a value greater than 1, and to model depression, a fractional value is used. Thus, the single parameter can be used to model diminished and enhanced reward perception in depression and mania respectively.

Social interactions in mania can be abnormal, such as acting in overly familiar ways with strangers^50^. Depressed agents on the other hand exhibit poor social relationships, with studies indicating lower scores for adequacy of both intimate and diffuse social relationships^32,91,92^. Studies also indicate elevated absenteeism at work, and lower work performance, in chronically-depressed individuals, and during depressed phases in BP patients^31^.

## Model of agent psychology and interactions

Consider two agents *A* and *B* paired in an interaction at time *t*. Now, agent *A*’s expectation of *B*’s level of cooperation in this interaction ($E_A(B)$) is the average of past levels of cooperation of *B*. *A* searches its memory for past experiences with *B*, which returns a set of relevant traces denoted by $L_e$.

$$\begin{aligned} E_A(B) = \frac{\sum _{i \in L_e} i[c]}{|L_e|}. \end{aligned}$$

Here, *i*[*c*] denotes the level of cooperation extended by the other player in the interaction that is described by the trace *i*. The primary assumption of this model, in line with studies^93,94^, is that *A* cooperates at the same level at which it expects *B* will cooperate.

1 $$\begin{aligned} c_A = E_A(B). \end{aligned}$$

This interaction induces an emotion in both agents, and consequently their moods and memory are updated as well. These will be explained in more depth in the following subsections. For the remainder of this section, we will continue with the same nomenclature of agents, *A* and *B* to maintain consistency and clarity.

### Interaction model

The agent society consists of four different types of agents: rational, manic, depressed, and bipolar agents. The set of all agents is denoted by $\mathscr {A}$. The society is modeled with a social network, and agents’ interactions are limited to their neighbors only. The social network is modeled as an undirected graph with agents as the nodes. Hence, the social network can be represented by

$$\begin{aligned} G&= (\mathscr {A},\mathscr {E})&\mathscr {E}&\subseteq \{(x,y)|(x,y) \in \mathscr {A}^2 \wedge x \ne y\}. \end{aligned}$$

The degree of the node determines the size of the neighborhood of an agent. The size of the neighborhood of the agent *A* is given by $\eta _A$, so 0 $\le \eta _A \le |\mathscr {A}|-1$.

As discussed in "Types of mood disorders", the size of the neighborhood of an agent is dependent on the type of the agent. The exact relationship between these is explained in "Modeling mood disorders".

The simulation consists of multiple rounds of interactions, and all agents are not active in all the rounds. At any such round *t*, an agent *A* is randomly paired with another agent *B* if and only if $(A,B) \in \mathscr {E}$ and both *A* and *B* are active at *t*.

An interaction between a pair of agents is based on the Continuous Prisoners Dilemma (CPD)^95,96^. In the standard Iterated Prisoners’ Dilemma, agents are restricted to only two actions—cooperate or defect. However, not all interactions can be realistically modeled with such restricted behavior.

In the CPD, agents can cooperate at any level between 0 and 1, where 0 and 1 correspond to the cases of completed defection and cooperation respectively. The concept and related payoff structure are adopted from Verhoeff^95^.

For the two agents *A* and *B*, their cooperation levels are denoted by $c_A$ and $c_B$, respectively. The payoff functions are derived from the discrete payoff matrix by linear interpolation^95^:

2 $$\begin{aligned} p_A(a,b)=c_Ac_BC + c_A\bar{c_B}S + \bar{c_A}c_BT + \bar{c_A}\bar{c_B}D, \end{aligned}$$

where *C*, *T*, *D*, *S* are the payoffs in the standard PD as shown below.
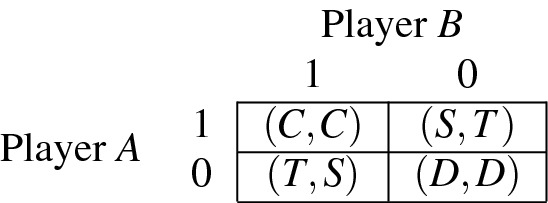


The values are chosen so $2C>T+D$ and $T>C>D>S$ (these are standard conditions applicable to the Prisoners’ Dilemma).

### Emotion and mood

We adopt the appraisal theory of emotion, as discussed in "Emotions and memory". The agent *A* evaluates the reward, which is the level of cooperation $c_B$ extended by *B* with respect to its expectation $E_A(B)$. The emotion of the agent *A* at time *t*, $\delta _A^t$ is therefore the difference between reward and expectation, given by

3 $$\begin{aligned} \delta _A^t = c_B - E_A(B) = c_B - c_A. \end{aligned}$$

By Eq. (1), $E_A(B) = c_A$ and thus the induced emotion is equivalent to the difference between the levels of cooperation of *A* and *B*. As discussed in "Interaction model", levels of cooperation $c_A$ and $c_B$ range from 0 to 1. Therefore Eq. (3) shows that -1 $\le \delta _A^t \le 1$. If $c_B > c_A$, it implies *A* has received a reward greater than it expected, which results in a positive emotion, $\delta _A^t$ > 0.

The mood $\nu _A^t$ of agent *A* is updated as follows.

4 $$\begin{aligned} \nu _A^t = \tanh {(\nu _A^{t-1} + \kappa _A(\delta _A^{t} - \nu _A^{t-1}))}. \end{aligned}$$

Here, $\kappa _A$ is the rate of change of mood and $0 \le \kappa _A \le 1$. When $\kappa _A$ is 1, the agent’s mood is fleeting and is the same as its emotion. But, when $\kappa _A$ is 0, the agent’s mood is constant and remains unperturbed by the interactions. To maintain symmetry, the mood is transformed to lie between − 1 and 1 using a sigmoidal function such as the hyperbolic tangent function.

### Memory encoding

The agent stores its experience after each interaction in its memory. This process of encoding leaves behind a trace, with initial strength $\phi _0$. We model that the encoding function is solely based on the emotional value of the interaction. As discussed in "Emotions and memory" and the references therein, it is clear that any encoding function satisfies the following two properties. First, emotionally charged events must be more strongly encoded than emotionally neutral events. Second, any agent can either be positively or negatively biased and the encoding function should allow this. After the interaction between *A* and *B*, *A* now encodes this experience into its memory, and the initial strength $\phi _0$ of this trace is given by:

5 $$\begin{aligned} \phi _0 = {\left\{ \begin{array}{ll} (-k_N \times \delta _A^t) + c &{} \delta _A^t \le 0 \\ (k_P \times \delta _A^t) + c &{} \delta _A^t > 0 \end{array}\right. } \end{aligned}$$

The negative and positive gradients are denoted by $k_N$ and $k_P$. It can be seen from Eq. (5) that $c \le \phi _0 \le \max {(k_N,k_P)}$. The values of the gradients may differ across agents, but for any agent it remains constant. The emotional bias of an agent, denoted by $\xi$, is a ratio of the two gradients.

6 $$\begin{aligned} \xi = \frac{k_P}{k_N}. \end{aligned}$$

If $\xi > 1$, then the agent is positively biased; it is negatively biased if $\xi < 1$, and has no emotional bias otherwise.

### Rate of forgetting

A trace cannot remain at the same strength as time progresses. We use an exponential forgetting curve as in many prior works^44–46^.

7 $$\begin{aligned} \phi _t = \phi _0 e^{-\theta _A t}. \end{aligned}$$

Here, $\theta _A$ is the rate of forgetting and varies across agents, but remains constant for an agent; $\theta > 0$. Each agent also has a minimum threshold on strength of traces, $\phi _{\min }$. When the strength of a trace drops below this threshold, it is deleted from memory.

However, when a trace is successfully retrieved from memory, its strength is reset to $\phi _0$.

**Remark 1 **The duration *d* of availability of a trace, without any refreshing, increases with initial strength, and decreases with the rate of forgetting.

For a trace to get deleted from memory, its strength should be less than the minimum threshold, i.e., $\phi _{d} < \phi _{\min }$. This means $\phi _0 e^{-\theta d} < \phi _{\min }$, which in turn yields:

8 $$\begin{aligned} d > \frac{1}{\theta } \ln \left( \frac{\phi _0}{\phi _{\min }}\right) . \end{aligned}$$

Since $\phi _0$ is higher for emotionally charged events by Eqs. (5),  (8) shows that emotionally charged events last longer in the memory.

### Mood and memory retrieval

Agent *A* searches its memory for traces pertaining to *B*, and obtains the filtered set of traces, $\mathbf {L}$. Mood dependent retrieval is the phenomenon where traces whose emotional states match the current mood are more likely to be retrieved. Since mood $\nu _A^t$ is a continuous variable such that $-1 \le \nu _A^t \le 1$ by Eq. (4), retrieving traces whose emotional states are exactly equal to the current mood is not feasible. Therefore, we use a triangular function (similar to the triangular apodization function used in signal processing and spectroscopy^97^), centered at the agent’s current mood, with a parameter $\gamma _A \ge 0$, to assign the likelihood of retrieval of each trace. Any function chosen should satisfy the following two requirements. First, for lower values of $\gamma _A$, the retrieval should not be selective, i.e., all traces should be retrieved irrespective of emotional state. Second, for higher values of $\gamma _A$, the retrieval chances should be better for those traces whose emotional state match the current mood. A triangular function satisfies both these requirements.

From the filtered set $\mathbf {L}$, the likelihood of retrieval of each trace is given by:

9 $$\begin{aligned} \forall j \in \mathbf {L}, p_j = \max (0,1 - \gamma _A|j[e] - \nu _A^t|). \end{aligned}$$

**Figure 1:**
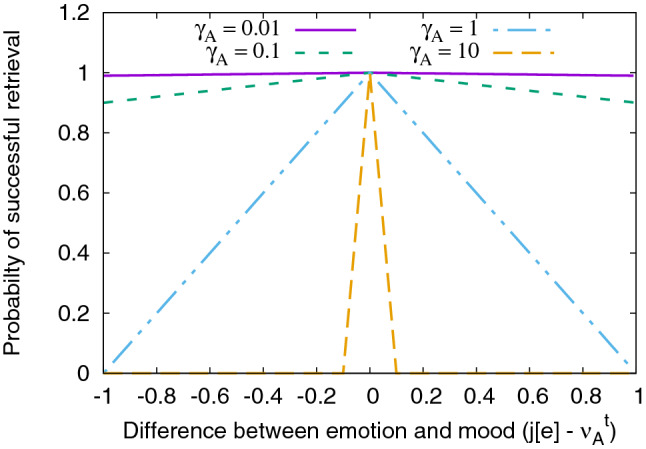
Mood dependence.

Here, emotion of trace *j* is denoted by *j*[*e*] and by Eq. (9), it is clear that $0 \le p_j \le 1$. When $\gamma _A$ is lower, the curve is flatter, and almost all traces are equally likely to be retrieved with probability tending to 1, which is the case of weak mood dependence. On the other hand, if *A* has a strong mood dependence, i.e., high $\gamma _A$, only traces whose emotional states are close to the current mood are retrieved. This is also seen in Fig. 1, where the curves corresponding to lower values of $\gamma _A$ are almost horizontal and assign probabilities close to 1 for all traces. On the other hand, the curves corresponding to higher values of $\gamma _A$ assign a probability of 0 to traces whose emotions do not match the current mood. Traces in $\mathbf {L}$ are probabilistically retrieved based on Eq. (9), and those traces that are successfully retrieved are returned as $L_e$.

### Modeling mood disorders

We model three different mood disorders: mania, depression, and bipolar disorder.

#### Mania and depression

As discussed in "Types of mood disorders", one of depression’s common symptoms is anhedonia^51^, the inability or the diminished ability to enjoy pleasurable events; in mania, the opposite is true^90^. Huys et al.^19^ used reward sensitivity to model perceived rewards to be different from actual rewards in individuals with mood disorders. In Eq. (3), we assume no difference between perceived and actual rewards, both equal to $c_B$. To accommodate manic and depressed agents, Eq. (3) is modified as:

10 $$\begin{aligned} \delta _A^t = \tanh {(\beta _A^t c_B - c_A)}. \end{aligned}$$

Here, $\beta _A^t$ is the reward sensitivity of agent *A* at time *t*, which remains constant for each agent, of either manic and depressed type. If agent *A* is depressed, then $0 \le \beta _A \le 1$. If *A* is manic, then $\beta _A > 1$. When $\beta _A$ is exactly 1, Eq. (10) reduces to Eq. (3), and *A* is a rational agent. Although theoretically $0 \le \beta _A < \infty$, for the sake of simulation a maximum value of $\beta _A$ is considered, and denoted by $\beta _{\max }$.

Depression is associated with reward hyposensitivity and anhedonia^89,98^, while mania is associated with excessive motivation^99^. Therefore we model reward sensitivity as a constant, trait-like parameter (to address hyposensitivity) with a low value (for anhedonia) for depressed agents, and a high value (for the opposite, in case of manic agents).

The mood $\nu _A^t$ is initialized with a value based on $\beta _A^0$ as:

11 $$\begin{aligned} \nu _A^0 = {\left\{ \begin{array}{ll} \beta _A^0 - 1 &{} 0 \le \beta _A^0< 1 \\ 1 + \frac{\beta _A^0 - 1}{\beta _{\max } - 1} &{} 1 < \beta _A^0 \le \beta _{\max } \end{array}\right. } \end{aligned}$$

It can be seen from Eq. (11), depressed agents are initialized with a negative mood, and manic agents are associated with a positive mood. $0 \le \nu _A^t \le 1$ for manic agents and $-1 \le \nu _A^t \le 1$ for depressed agents.

In the case of depression, it has been seen that depressed people are more likely to be connected to other depressed individuals^100^. In line with this, we introduce a connection diversity parameter for depressed agents only, denoted by $\pi$. If $\pi$ is 0.5, that implies half the connections of the agent are depressed agents, and the remaining connections are distributed among the other types equally.

#### Bipolar disorder

Bipolar disorder (BP) is a mood disorder where the agent’s mood oscillates between mania and depression. In line with existing rhythmic models^34,81,101^ that assume mood swings as periodic, we model mood fluctuation in BP using a sine curve with the period varied periodically by the parameter $\omega _A$ and a constant offset $\lambda _A$. The mood of a bipolar agent *A* at any time *t* is given by:

12 $$\begin{aligned} \nu _A^t = \sin {\omega _A t} + \lambda _A. \end{aligned}$$

The offset $\lambda _A$ determines if the agent is mostly manic or mostly depressed and $-1 \le \lambda _A \le 1$. If $\lambda _A > 0$, the agent is mostly manic it is mostly depressed if $\lambda _A < 0$. The parameter $\omega _A$ determines the period of cycling. Clinical studies suggest there are phases of rapid cycling in BP patients^82,83^, and that the rate of cycling is different across individuals. In our model, $\omega _A$ is updated every 50 rounds, and it is sampled from an exponential distribution with mean $\omega '_A$, which remains constant for an agent.

In bipolar agents, the perceived reward is dependent on the agent’s mood^26,102^, and hence cannot be modeled by a constant reward sensitivity. Instead, a time-varying reward sensitivity, $\beta _A^t$ is used to model bipolarity.

13 $$\begin{aligned} \beta _A^t = {\left\{ \begin{array}{ll} 1 &{} \nu _A^t = 0 \\ \nu _A^t + 1 &{} -1 \le \nu _A^t< 0 \\ 1 + \nu _A^t(\beta _{\max } - 1) &{} 0 < \nu _A^t \le 1 \end{array}\right. } \end{aligned}$$

The emotion induced by an interaction for bipolar agents is given by Eq. (10). The only difference is that, the reward sensitivity $\beta _A^t$ is constant for manic and depressed agents, but changes with time for bipolar agents. It is clear from Eq. (13), that the agent overestimates reward when manic, and perceives diminished rewards when depressed.

#### Social interactions and mood disorders

Another symptom associated with manic and depressed phases is the relatively higher and lower drive of the individual. We already alluded to this concept in "Interaction model", stating agents can choose if they want to participate or not in any round of interaction. This is modeled as drive of an agent $\zeta _A$, with $0 \le \zeta _A \le 1$. In each round, the agent decides to participate with probability $\zeta _A$, given by:

14 $$\begin{aligned} \zeta _A = {\left\{ \begin{array}{ll} 0.5 &{} \beta _A^0 = 1 \\ 0.5\beta _A^0 &{} 0 \le \beta _A^0< 1 \\ 0.5 + 0.5\frac{\beta _A^0 - 1}{\beta _{\max } - 1} &{} 1 < \beta _A^0 \le \beta _{\max } \end{array}\right. } \end{aligned}$$

The size of *A*’s neighborhood, $\eta _A$ is also vastly different for manic and depressed agents with depressed agents being more lonely and having smaller personal network^50,91^. The social reach is a function of reward sensitivity, and is given by:

15 $$\begin{aligned} \eta _A = {\left\{ \begin{array}{ll} 0.5|\mathscr {A}| &{} \beta _A^0 = 1 \\ 0.5\beta _A^0|\mathscr {A}| &{} 0 \le \beta _A^0< 1 \\ \left( 0.5 + 0.5\frac{\beta _A^0 - 1}{\beta _{\max } - 1}\right) |\mathscr {A}| &{} 1< \beta _A^0 < \beta _{\max } \end{array}\right. } \end{aligned}$$

Both these parameters are constant for an agent, and Eqs. (14) and  (15) are obtained by applying min–max rescaling to transform the ranges. For example, in the case of depressed agents, $0 \le \beta _A^0 \le 1$ and $0 \le \zeta _A < 0.5$. Hence, min–max rescaling is used to transform the value of $\beta _A^0$ to an appropriate value of $\zeta _A$.

The mood is a complex phenomenon^103^. Also, there is no direct relationship between mood and behavior^104^. It is fuzzy, ambiguous, could be fleeting or lasting, and a person cannot pinpoint the “why” of their current mood^105^. There is also no direct causal relationship between mood and the drive to participate in an activity^105^. Therefore, there is no precise way to model drive and network size as state-like factors dependent on current mood.

## Agent types and design

An agent society comprising four different types of agents is considered. The agents are categorized into these types by behavior, decision making and internal attributes. The system is initialized with various specifications such as the number of agents, proportion of various types, and the number of rounds of interaction. The set of all agents is denoted by $\mathscr {A}$ and the number of rounds is denoted by *K*. In the following subsections, we will define the attributes of an agent and the different types of agents.

### Agent attributes

An agent *A* has several attributes that determine its nature and behavior. First, *A* has an unique identifier, denoted by $\alpha _A$. The total number of agents in the system is $|\mathscr {A}|$, therefore $\alpha _A \in \{1,2, \ldots ,|\mathscr {A}|\}$. The performances of agents are compared based on cumulative payoffs at the end of the simulation, and this is captured by a non-negative real number. The mood of *A*, $\nu _A^t$ and the rate of change of mood $\kappa$, are as described in "Mood and memory". The reward sensitivity $\beta _A^t$ is determined by its type, as discussed in "Mood disorders". The size of *A*’s neighborhood $\eta _A$ is determined by the initial reward sensitivity $\beta _A^0$ as given by Eq. (15). The drive of *A* $\zeta _A$, a measure of the level of engagement, is also a function of $\beta _A^0$ and is given by Eq. (14). *A* also maintains a set of its neighbors, $\mathbf {N}_A$.

The memory of *A* has five attributes. The rate of forgetting $\theta _A$ and the minimum threshold of trace strength $\phi _{\min }$, are as in "Forgetting". Another attribute is the level of mood dependence used to model mood-dependent retrieval in Eq. (9). It also needs to maintain a pair of gradients $(k_N,k_P)$ which are used as positive and negative gradients in the encoding function (Eq. 5). Lastly, the memory maintains a set of traces.

Each trace contains information pertaining to a single interaction, such as the identifier of the counterpart agent involved and the level of cooperation extended. Other information include the emotion $\delta _A^t$ induced by the interaction (as given by Eqs. (3) and (10)). The trace is encoded and assigned an initial strength $\phi _0$ (as given by Eq. (5)) and this is also captured. The strength of the trace, $\phi _t$ decays with time (as given by Eq. (7)).

### Agent types

As discussed in "Model of agent psychology and interactions", we consider four different types of agents: rational, manic, depressed, and bipolar. Table 1 summarizes the key differences among them.

**Table 1:** Summary of agent types and attributes.

Attribute	Rational	Manic	Depressed	Bipolar
$\beta _A^0$	1	[1,10]	[0,1]	[0,10]
$\nu _A^0$	0	[0,1]	[− 1,0]	[− 1,1]
$\kappa _A$	0	0.1	0.1	N/A
$\eta _A$	$0.5|\mathscr {A}|$	$[0.5|\mathscr {A}|,|\mathscr {A}|]$	$[0,0.5|\mathscr {A}|]$	$[0,|\mathscr {A}|]$
$\zeta _A$	0.5	[0.5,1]	[0,0.5]	[0,1]

#### Rational agents

A rational agent is considered the baseline. It perceives rewards as they are, hence has a reward sensitivity of exactly 1, and its mood always stays neutral at 0. The size of its neighborhood is exactly half the total number of agents in the system (Eq. 15). Similarly, its drive is also 0.5, based on Eq. (14). This agent type is based upon the ideal of a perfect rational human.

#### Manic agents

A manic agent is the virtual equivalent of a human with chronic mania. For simulation, the value of $\beta _{\max }$ is taken to be 10 and hence, the reward sensitivity lies between 1 and 10 (see "Mood disorders"). Mood $\nu _A^t$ is initialized with a positive value based on $\beta _A$ by Eq. (11), and the mood always remains positive. Manic agents exhibit greater drive than rational agents, and therefore $\zeta _A > 0.5$ by Eq. (14). Manic agents’ higher levels of social engagement are modeled with larger neighborhoods, from Eq. (15).

#### Depressed agents

A depressed agent is based on humans with Major Depressive Disorder. The reward sensitivity of these agents lies between 0 and 1 (see "Mood disorders"). Mood $\nu _A^t$ is initialized with a negative value based on $\beta _A$, by Eq. (11), and the mood always remains negative. Depressed agents possess smaller neighborhoods as given by Eq. (15). Their levels of engagement are also lower, modeled with a lower drive than rational agents in Eq. (14).

#### Bipolar agents

A bipolar agent is subject to oscillating periods of mania and depression. As discussed in "Mood disorders", mood updates are modeled by a sine function. Bipolar agents have two additional attributes apart from the basic attributes defined in "Agent attributes": $\omega _A$, which controls the rate of cycling, and the offset $\lambda _A$.

The sizes of the neighborhood and the drive of bipolar agents are given by Eqs. (15) and (14), respectively. Since mood updates happen intrinsically due to internal rhythms according to Eq. (12), the rate of mood change $\kappa _A$ is not pertinent for bipolar agents.

A summary of all four agent types and the permissible ranges for their attributes is given in Table 1.

## Results

A system of 200 agents with representation from all four types is simulated for 2000 rounds of interaction. As discussed in "Agent types", the parameters of each agent are assigned in the range of permissible values. In each round, agents are paired according to the interaction model outlined in "Interaction model". The agents interact, update their payoffs, emotion, mood, and their availability for the next round. Different parameters of agents are varied in each experiment and their impacts on total payoff are presented in the subsequent subsections.

**Figure 2:**
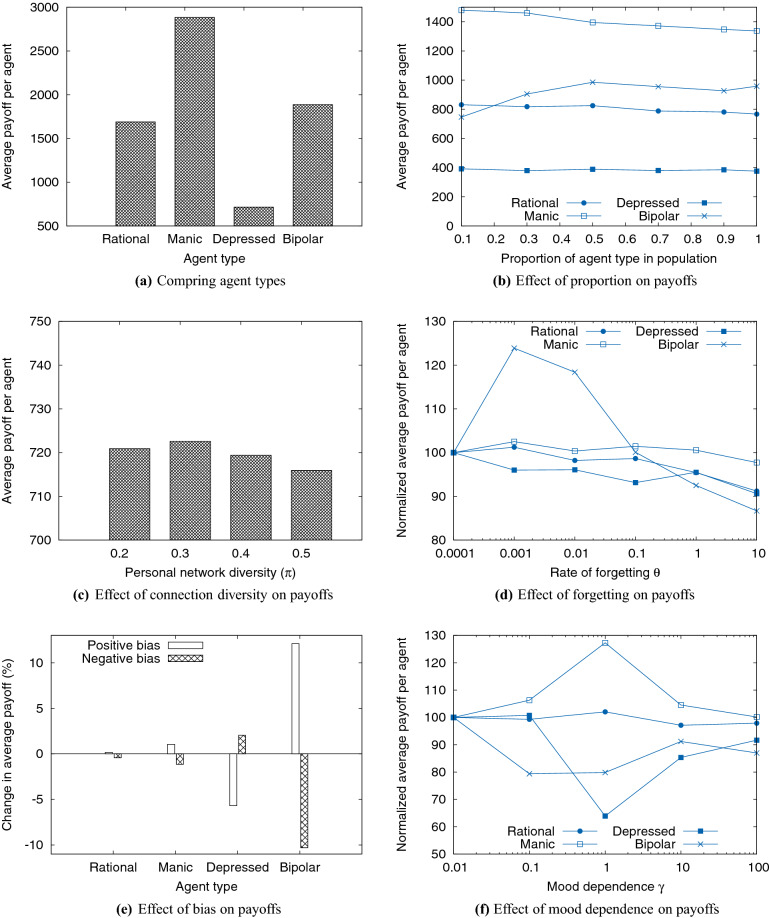
Results.

### Comparing payoffs of all agent types

For this experiment, all four types of agents are equally represented (25%). We find that payoffs are highest for manic agents and least for depressed agents (Fig. 2a). Although bipolar agents show vastly different payoffs between themselves, on average the payoff of bipolar agents is greater than that of rational agents by about 10%. It is also seen that the average payoff of manic agents is about 70% higher than the average payoff of rational agents. When compared with rational agents, an average depressed agent’s payoff is roughly 60% lower.

### Effect of varying the population composition

In this experiment, for each type of agent, different levels of representation in the total population are considered, with the remaining types distributed equally. For example, if we consider 10% representation of rational agents in the system, then the remaining 90% is split between the other three types equally. In Fig. 2b, each curve depicts the effect on the average payoff of that type when the proportion of agents of that type is varied. No significant difference in the level of payoff is observed for rational and depressed agents when their proportion in the system is increased. However, increasing the proportion of manic agents resulted in a significant dip in their average payoff. For bipolar agents, a higher value of average payoff is observed with an increasing proportion of the corresponding type.

### Effect of diversity of connections in depressed agents

In this experiment, we have an agent society with equal numbers of agent in all four types, and we vary the connection diversity of depressed agents from 0.2 to 0.5. A connection diversity of 0.25 implies there is an equal number of agents from all four types in the personal network of the agent. As seen in Table 1, the highest value of $\eta$ for a depressed agent is $0.5|\mathscr {A}|$. If $\pi$ > 0.5, then the number of connections with depressed agents would exceed $0.25|\mathscr {A}|$. This is not possible because the total number of agents of each type is $0.25|\mathscr {A}|$. Through simulation, it is seen that a moderate value of $\pi$ brings out the highest average payoff (Fig. 2c).

### Effect of forgetting

In this experiment, the rate of forgetting is varied in powers of 10 starting from $10^{-4}$ until 10. To avoid potential confounders, other parameters such as the positive and negative gradients, mood dependence, etc., are fixed. The value of gradients for the encoding function (Eq. 5), $k_N,k_P$ and the intercept *c* are chosen such that the initial strength of traces are less than 100. From Eq. (8), the minimum value of *d*, the duration of availability with $\theta _A = 0.0001$ is much greater than 2000, which is the total number of interactions. Hence this corresponds to the case of a perfect memory, where an agent remembers every single interaction right from the beginning. On the other hand, when $\theta _A = 10$, the maximum duration of availability is less than 1, which implies the agent has no memory at all. Through simulation, it is observed that a stronger retention, i.e., a lower value of $\theta _A$ offers better payoffs than weaker retention (Fig. 2d). However, the exact difference varies across types. Among manic agents, high values of $\theta _A$ cause 4% lower payoffs than agents with perfect memory. Among rational and depressed agents, those with no memory have an average payoff that is roughly 10% lower than agents with perfect memory. Among bipolar agents, agents with a near-perfect memory perform better than those with either a perfect memory or no memory.

### Effect of emotional bias

Considering a system with equal proportions of all agent types, with other parameters such as $\theta _A$ and $\gamma _A$ fixed to avoid any potential confounders, the average payoff is considered the baseline (0), and the payoff associated with both biases are plotted as percentage differences from the baseline (Fig. 2e). In manic, and bipolar agents, positive bias is associated with higher payoffs, with positively biased agents performing about 12% better among bipolar agents, but only roughly 1% better among manic agents. There is not much change for rational agents. However, among depressed agents, negatively biased agents perform roughly 2% better than average.

### Effects of mood-based retrieval

To analyze the effects mood dependence, we considered values from $10^{-2}$ to 100 in powers of 10. For each type, the average payoff at the lowest level of mood dependence (0.01) is considered 100 and the payoffs at other levels are scaled appropriately. Fig. 2f shows that mood dependence does not have any significant effect on rational agents. However, for manic agents an intermediate value of mood dependence offers the highest payoff ($\sim$ + 30%) while in depressed agents, an intermediate value of mood dependence gives the lowest payoff ($\sim - 40$%). In bipolar agents, low mood dependence maximizes payoff.

### Effect of reward sensitivity

**Figure 3:**
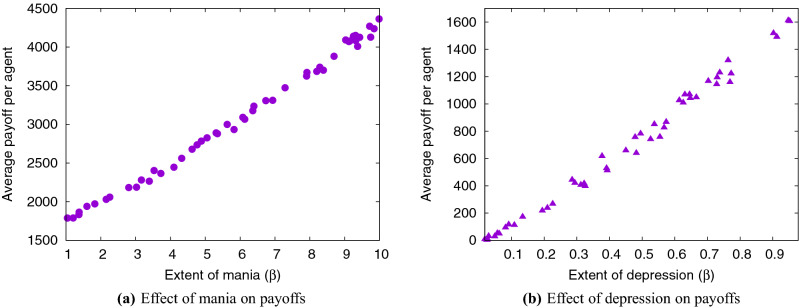
Effect of levels of mania and depression.

As discussed in "Mood disorders", the reward sensitivity $\beta _A$ is a measure of the extent of mania or depression. Figure 3a depicts the almost-linear relationship between $\beta _A$ and average payoff. It is observed that payoffs increase with increasing mania, which is fairly obvious because the higher the mania, higher is the drive in our model (Eq. 14). An almost-linear relationship between $\beta _A$ and payoff is observed in depressed agents also (Fig. 3b). The observed relationship between payoffs and level of mania/depression is in line with prior clinical studies^106,107^, but there is no obvious analytical explanation in those. This model thus allows for insights into the observed outcomes. It is also clear that depressed agents at any level perform worse than manic agents.

## Conclusion

Mood disorders are unfortunately prevalent in society, hence models that capture them realistically are essential and relevant. We present a model that captures some important aspects of social interactions and success, given mood disorders and memory biases. A linear encoding function with different gradients for positive and negative emotions is used to model the relationship between emotion and memory. Mood-dependent memory retrieval is modeled using a triangular function centered at the agent’s current mood. Social interactions are considered in mood disorders, as opposed to most prior work that considered agents in isolation.

Based on simulation of an agent society with different types of agents, we obtain results about relative payoffs and other aspects that are in agreement with, and extend, published studies. Our results concur with psychological studies that establish a relationship between severity of depression and lower performance^106^. Clinical studies also suggest diminished performance in depressed individuals and improved performance in cases of mania^107–109^, as also seen in our model.

Mood-dependent memory retrieval remains unexplored in prior works and clinical studies, and there seem to be no models that establish its impact on performance. We can show that an intermediate level of mood dependence brings the highest payoffs for manic agents, and lowest payoff for depressed agents. Our work can thus also be extended to effects of various traits or biases, some of which may not be easy to study empirically.
